# A Highly Unusual V1 Region of Env in an Elite Controller of HIV Infection

**DOI:** 10.1128/JVI.00094-19

**Published:** 2019-05-01

**Authors:** Zachary A. Silver, Gordon M. Dickinson, Michael S. Seaman, Ronald C. Desrosiers

**Affiliations:** a Medical Scientist Training Program, Miller School of Medicine, University of Miami, Miami, Florida, USA; b Department of Pathology, Miller School of Medicine, University of Miami, Miami, Florida, USA; c Infectious Diseases Section, Miami Veterans Affairs Health Care System, University of Miami, Miami, Florida, USA; d Department of Medicine, Miller School of Medicine, University of Miami, Miami, Florida, USA; e Center for Virology and Vaccine Research, Beth Israel Deaconess Medical Center, Boston, Massachusetts, USA; Emory University

**Keywords:** elite controller, HIV-1, V1 domain, bNAb

## Abstract

HIV elite controllers represent a remarkable minority of patients who maintain normal CD4^+^ T-cell counts and low or undetectable viral loads for decades in the absence of antiretroviral therapy. To examine the possible contribution of virus attenuation to elite control, we obtained a primary HIV-1 isolate from an elite controller who had been infected for 19 years, the last 10 of which were in the absence of antiretroviral therapy. Full-length sequencing of this isolate revealed a highly unusual V1 domain in Envelope (Env). The V1 domain in this HIV-1 strain was 49 amino acids, placing it in the top 1% of lengths among the 6,112 Env sequences in the Los Alamos National Laboratory online database. Furthermore, it included two additional N-glycosylation sites and a pair of cysteines suggestive of an extra disulfide loop. Virus with this Env retained good infectivity and replicative capacity; however, analysis of recombinant viruses suggested that other sequences in Env were adapted to accommodate the unusual V1 domain. While the long V1 domain did not confer resistance to neutralization by monoclonal antibodies of the V1/V2-glycan-dependent class, it did confer resistance to neutralization by monoclonal antibodies of the V3-glycan-dependent class. Our findings support results in the literature that suggest a role for long V1 regions in shielding HIV-1 from recognition by V3-directed broadly neutralizing antibodies. In the case of the elite controller described here, it seems likely that selective pressures from the humoral immune system were responsible for driving the highly unusual polymorphisms present in this HIV-1 Envelope.

**IMPORTANCE** Elite controllers have long provided an avenue for researchers to reveal mechanisms underlying control of HIV-1. While the role of host genetic factors in facilitating elite control is well known, the possibility of infection by attenuated strains of HIV-1 has been much less studied. Here we describe an unusual viral feature found in an elite controller of HIV-1 infection and demonstrate its role in conferring escape from monoclonal antibodies of the V3-glycan class. Our results suggest that extreme variation may be needed by HIV-1 to escape neutralization by some antibody specificities.

## Abstract (precis)

Elite controllers have long provided an avenue for researchers to reveal mechanisms underlying control of HIV-1. While the role of host genetic factors in facilitating elite control is well known, the possibility of infection by attenuated strains of HIV-1 has been much less studied. Here we describe an unusual viral feature found in an elite controller of HIV-1 infection and demonstrate its role in conferring escape from monoclonal antibodies of the V3-glycan class. Our results suggest that extreme variation may be needed by HIV-1 to escape neutralization by some antibody specificities.

## INTRODUCTION

Nearly 40 years after its discovery, human immunodeficiency virus (HIV) continues to spread at an alarming rate (1). Although antiretroviral therapy (ART) dramatically reduces HIV transmission from and morbidity among infected individuals, access and adherence to ART can be challenging. In the absence of treatment, HIV-1 infection progresses to the development of AIDS in >99% of cases (2, 3). Elite controllers represent a remarkable minority of patients who maintain normal CD4^+^ T-cell counts and low or undetectable viral loads in the absence of ART (4). Such patients provide researchers with a unique opportunity to study the mechanisms underlying AIDS progression in individuals chronically infected with HIV (5).

Investigations of elite controllers have primarily focused on elucidating the host determinants of elite control (6–10). Less studied, however, is the extent to which rare, highly unusual HIV polymorphisms may contribute to elite control (11–14). Highly unusual, attenuating polymorphisms in the viral genome can arise in the presence of immune pressure as a means to escape that pressure (15, 16). The characterization of HIV epitopes targeted by protective cytotoxic T-cells (CTLs) and escape mutants that attenuate circulating virus has provided insights into regions of the HIV genome that are important for continuous replication of the virus at high levels (17). In addition, infection by a transmitted/founder virus containing an attenuating polymorphism that is resistant to reversion (such as HIV with an inactivating deletion in its *nef* gene) may also result in elite control (18, 19). The study of highly unusual, potentially attenuating viral polymorphisms, whether immune mediated or transmitted by the founder virus, in the context of HIV elite control can improve our understanding of HIV biology and shed light on viral determinants of AIDS pathogenesis.

In this report, we describe an elite controller (VA40774) from whom we isolated a group M, clade B replication-competent strain of HIV-1. Sequencing of the primary HIV-1 isolate from this patient revealed an elongation of the Envelope (Env) V1 domain that renders it the longest in the 2016 Los Alamos National Laboratory (LANL) Sequence Compendium and among the top 1% in the LANL online sequence database. When exchanged into other viral backbones, this V1 domain causes an attenuation of viral infectivity. We show that the presence of this long V1 domain is sufficient for either partial or complete escape from V3-glycan-targeting broadly neutralizing antibodies (bNAbs) 10-1074 and PGT121 but not by members of other classes of bNAbs.

## RESULTS

### Study subject.

**FIG 1:**
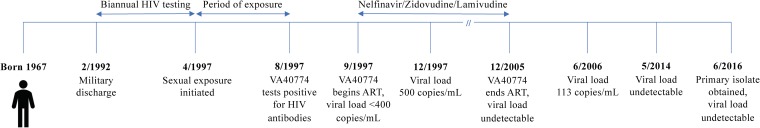
Timing of HIV-1 infection in patient VA40774. VA40774 tested positive for HIV-1-specific antibodies in August 1997 and began treatment with nelfinavir and zidovudine/lamivudine shortly thereafter. At the time of ART initiation, the patient had a viral load of <400 RNA copies per ml of plasma. The patient’s viral load reached 500 copies of viral RNA per ml of plasma 3 months following the initiation of ART but remained negative for the following 8 years of treatment. Seven months after therapy was discontinued, the patient’s viral load reached 113 copies of viral RNA per ml of plasma. Since this time, the viral load has remained under the limit of detection (<50 copies of viral RNA per ml of plasma). The patient’s CD4^+^ T-cell counts have remained above 600 cells per µl of blood throughout the infection.

VA40774 is a male individual who began biannual HIV testing on 2 February 1992 following his departure from military service (Fig. 1). He first tested positive for HIV-specific antibodies on 19 August 1997 at the age of 30, approximately 4 months following initiation of sexual contact with an HIV-positive male who died from AIDS on 23 September 1997. Twenty days following his diagnosis, VA40774 was started on antiretroviral therapy consisting of nelfinavir (750 mg three times per day) and zidovudine/lamivudine (300/150 mg twice per day). Although the exact date of infection is unknown, the timing of his exposure and previously negative HIV tests indicate that VA40774 was infected between 17 and 130 days prior to the initiation of therapy. On the day that ART was started, the patient had a viral load of less than 400 copies of RNA per ml of plasma. In December 1997, 3 months after the initiation of ART, the patient had 500 copies of viral RNA per ml of plasma. Plasma levels of HIV RNA were undetectable during the following 8 years of treatment. The patient discontinued ART in December 2005 and HIV RNA was not detected in his plasma in the 11 years that followed, except for a single viral load measurement of 113 RNA copies per ml of plasma in June 2006. VA40774 remains positive for HIV-specific antibodies by Western blotting, and his partner is periodically checked but remains HIV negative despite unprotected sex over a 15-year period.

To explore the possibility that this case of elite control may have a genetic component, we determined the human leukocyte antigen (HLA), killer cell immunoglobulin-like receptor (KIR), and *ccr5* genotypes of VA40774 (Table 1). The patient’s class I HLA alleles consist of A*01, A*30, B*15, B*44, C*02, and C*04. The patient is positive for all KIR alleles except for 2DS2, 2DS3, and 3DS1 and is negative for the *ccr5Δ32* allele. VA40774 does not have any alleles that are strongly associated with elite control. However, HLA-B*44 has been shown to result in modestly lower viral set points and slow CD4^+^ T-cell decline in a cohort of individuals acutely infected with HIV-1 (20).

**TABLE 1:** HLA and KIR genotypes^*a*^

Subject	*ccr5Δ32* allele	HLA genotypes			KIR genotypes	
		HLA-A	HLA-B	HLA-C	Positive	Negative
VA40774	No	01:01:01:01, 30:01:01	15:47:01, 44:03:01	02:10, 04:01:01:01	2DL1, 2DL2, 2DL3, 2DL4, 2DL5, 2DS1, 2DS4, 2DS5, 3DL1, 3DL2, 3DL3, 2DP1, 3DP1	2DS2, 2DS3, 3DS1

a HLA, human leukocyte antigen; KIR, killer cell immunoglobulin-like receptor.

### Sequence analysis of VA40774’s HIV-1 isolate.

**FIG 2:**
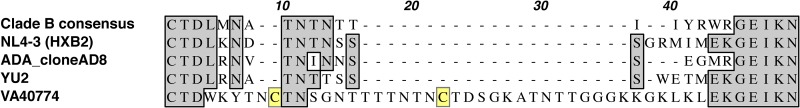
Comparison of Envelope sequences reveals a dramatic elongation of the V1 domain in an HIV-1 elite controller. T-Coffee alignment of VA40774’s Envelope V1 domain against the HIV-1 clade B consensus sequence, one CXCR4-tropic (NL4-3) virus, and two CCR5-tropic (ADA, YU2) viruses reveals the presence of an elongation in V1. The V1 domain in VA40774’s HIV Envelope contains two cysteines (yellow boxes) separated by 12 amino acids and has two additional sites for N-linked glycosylation (NXT/NXS). Amino acid positions are shaded in gray where an amino acid represents more than 51% of the variation for that position. Positions for which there is no consensus amino acid are left unshaded. Gaps in the amino acid sequence are shown with a dash (-).

Near full-length PacBio sequencing of the HIV-1 isolated from VA40774 in 2016 resulted in 12,831 reads of insert with a total of 36,704,432 sequenced HIV-1 nucleotides. These sequences were deconvoluted to reveal an essentially homogenous virus population belonging to group M, clade B. Analysis of the HIV-1 genome showed an extremely rare V1 domain in Envelope. Specifically, VA40774’s isolate contains an additional 25 amino acids compared to the HIV-1 clade B consensus V1 domain (Fig. 2). At 49 amino acids in length, this V1 domain is the longest of 181 group M HIV-1 sequences in the 2016 LANL Sequence Compendium and among the top 1% of the 6,112 group M Env sequences in the LANL online sequence database. The increased length of the V1 domain confers an additional two N-glycosylation sites and two cysteines. While the addition of N-glycosylation sites among HIV-1 strains with increasingly long V1 domains is common, the addition of cysteines is less commonly found among HIV-1 and more common in HIV-2 and simian immunodeficiency virus (SIV) (21, 22). van den Kerkhof et al. reported previously that only 4.7% of HIV-1 isolates contain two extra cysteines in V1, while four extra cysteines are present in only 0.1% of HIV-1 sequences (23). Thus, the Envelope V1 domain in VA40774's isolate is notably unique in both its length and cysteine content.

We separately analyzed the remaining eight VA40774 HIV-1 proteins for rare amino acid variants. The results of this analysis revealed a total of 14 amino acid substitutions across VA40774’s HIV-1 that are not found among any of the 181 group M HIV-1 sequences from the 2016 LANL Sequence Compendium. Our analyses indicate that such a frequency of rare mutations is standard fare for an individual HIV-1 isolate in the database. Unusual, difficult-to-revert polymorphisms such as deletions, insertions, or frameshift mutations were not observed in these eight open reading frames (ORFs).

### Infectivity of V1 domain recombinants.

**FIG 3:**
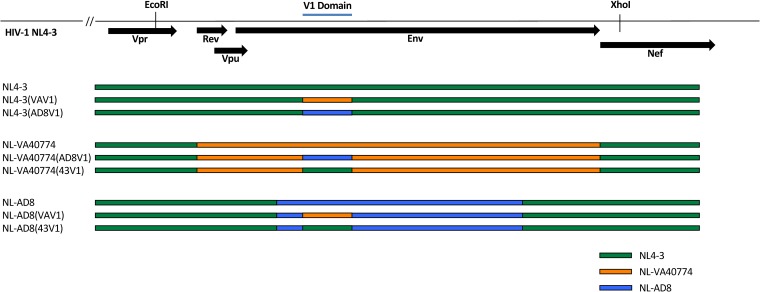
Schematic of recombinant viruses. HIV *env* sequences of NL4-3, NL-VA40774, and NL-AD8 were gene synthesized and subcloned to generate several recombinant viruses with different Envelopes and V1 domain sequences. Stretches of nucleotides belonging to NL4-3, NL-VA40774, and NL-AD8 are depicted in green, orange, and blue, respectively (43V1, V1 domain of NL4-3; AD8V1, V1 domain of NL-AD8; VAV1, V1 domain of NL-VA40774).

**FIG 4:**
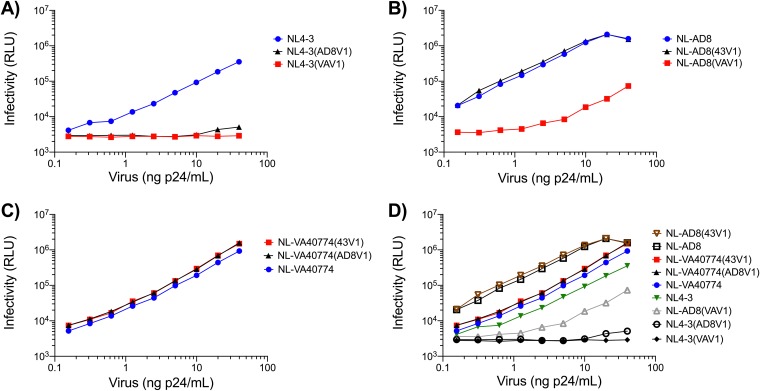
Comparative levels of infectivity. TZM-bl cells were infected with serial dilutions of each V1 domain recombinant, and infectivity was determined based on luciferase activity, or relative light units (RLU), after 48 h. (A) Exchange of the V1 domain from either NL-AD8 or NL-VA40774 into the NL4-3 background. (B) Exchange of the V1 domain from either NL4-3 or NL-VA40774 into NL-AD8. (C) Exchange of the V1 domain from either NL4-3 or NL-AD8 into NL-VA40774. (D) Compilation of data in panels A to C.

To determine the influence of a long V1 domain on HIV-1 particle infectivity, we generated several V1 domain recombinants on the NL4-3, NL-AD8, and VA40774 Env (NL-VA40774) backbones (Fig. 3). Infectivity was determined by exposing 10,000 TZM-bl cells to serial dilutions of each virus stock, incubating for 48 h, and measuring luciferase activity. NL-AD8 was the most infectious strain tested, followed by NL-VA40774 and NL4-3 (Fig. 4). While exchange of the V1 domain from NL4-3 into NL-AD8 had no marked effect on infectivity, exchange of NL-VA40774’s V1 domain into NL-AD8 resulted in a 2 log drop in infectivity. NL4-3 could not tolerate V1 domain exchanges, as seen by the complete abrogation of virus infectivity upon exchanging the wild-type NL4-3 V1 domain with the V1 domain of either NL-AD8 or NL-VA40774. Exchange of the V1 domain from NL4-3 or NL-AD8 into NL-VA40774 did not alter the overall infectivity of NL-VA40774. Thus, NL-VA40774 could well tolerate the exchange of V1 regions from the other strains but the other strains did not well tolerate the exchange of NL-VA40774’s V1 domain into them. The recombinant virus strains (listed from most infectious to least infectious) were as follows: NL-AD8(43V1), NL-AD8, NL-VA40774(43V1), NL-VA40774(AD8V1), NL-VA40774, NL4-3, NL-AD8(VAV1), NL4-3(AD8V1), and NL4-3(VAV1).

### Replicative capacity and tropism of V1 domain recombinants.

Given that modifications to the V1 domain may confer CXCR4 tropism (24–26), we investigated the replicative capacity and tropism of each parental strain and V1 recombinant.

**FIG 5:**
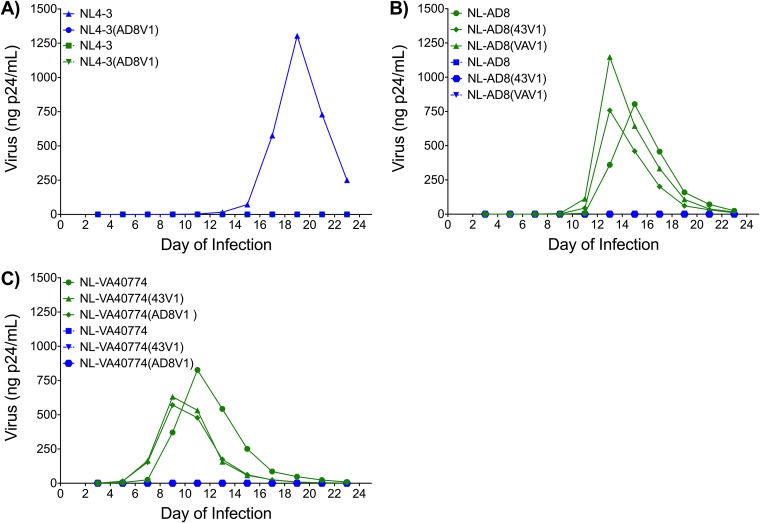
Replicative capacity and coreceptor usage of V1 recombinants. A modified SupT1 cell line expressing either CXCR4 (blue) or CCR5 (green) was infected with each V1 recombinant to determine the extent to which the V1 domain might alter replicative capacity and coreceptor usage. (A) Exchange of the V1 domain from NL-AD8 into NL4-3 was not compatible with virus replication. As expected, NL4-3 replicated only in the SupT1 cell line expressing CXCR4, with peak virus production on day 19. (B) Exchange of the V1 domain from NL4-3 or NL-VA40774 into NL-AD8 had minimal effect on NL-AD8 replication. The V1 domain recombinants NL-AD8(43V1) and NL-AD8(VAV1) reached peak virus production on day 13, 2 days earlier than wild-type NL-AD8. The V1 domain exchanges had no effect on the tropism of NL-AD8. (C) NL-VA40774 is a strictly CCR5-tropic isolate with replication kinetics that are minimally altered by V1 exchanges. The V1 domain recombinants NL-VA40774(43V1) and NL-VA40774(AD8V1) reached peak virus production on day 9, in contrast with the parental NL-VA40774, whose production peaked on day 11. Exchange of the V1 domains from either NL4-3 or NL-AD8 did not affect the tropism of NL-VA40774.

NL4-3, NL-AD8, and NL-VA40774 all replicated well (Fig. 5). Consistent with the TZM-bl infectivity data, there was no replication of the NL4-3(AD8V1) recombinant. NL-AD8, however, replicated well regardless of whether the V1 domain originated from NL4-3 or NL-VA40774. As expected, the NL4-3 strain replicated only in SupT1 cells expressing CXCR4 (Fig. 5A), while the NL-AD8 strain replicated only in the CCR5^+^ SupT1 cells (Fig. 5B). NL-VA40774 and the NL-VA40774 V1 exchange recombinants replicated only in the CCR5-expressing cell line (Fig. 5C). Thus, exchange of the V1 domains did not have an effect on the tropism of the parental virus (Fig. 5).

### Neutralization characteristics of VA40774’s HIV-1 Envelope.

**FIG 6:**
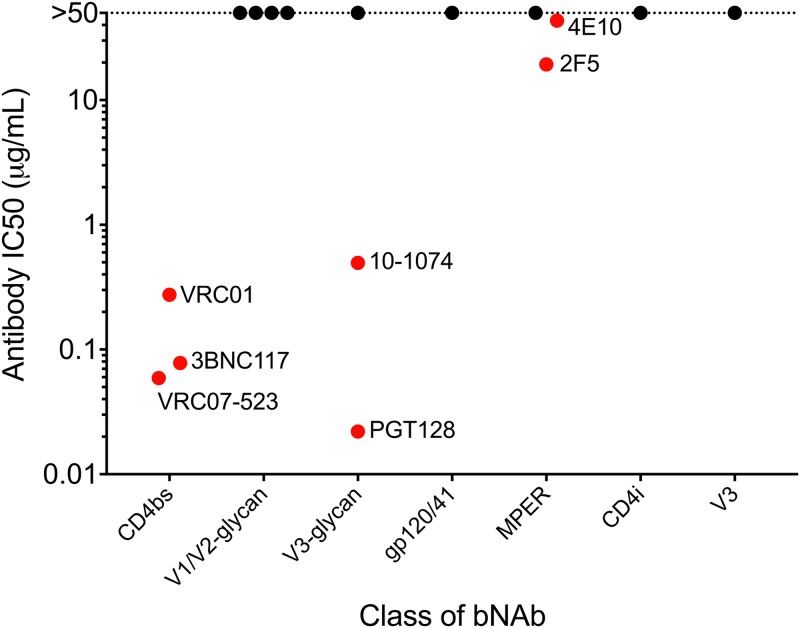
Neutralization characteristics of *env* recombinant. NL-VA40774 demonstrated sensitivity to bNAbs targeting the CD4bs, V3-glycans, and MPER, but interestingly was resistant to all V1/V2-glycan bNAbs. The concentrations of bNAb required for 50% neutralization of NL-VA40774 (IC_50_) are shown in red, and IC_50_ values of >50 µg/ml are shown in black. The V3-glycan antibody PGT128 was the most potent inhibitor of NL-VA40774, followed by CD4bs antibodies VRC07-523, 3BNC117, and VRC01. Continuing in order of decreasing potency, V3-glycan antibody 10-1074 is followed by MPER antibodies 2F5 and 4E10. The following antibodies did not neutralize NL-VA40774 (i.e., had IC_50_s above 50 µg/ml): PG9, PG16, PGDM1400, CAP256-VRC26.25, PGT121, 8ANC195, 10E8, 17b, and 447-52D.

Given that antibody responses can drive evolutionary changes within the V1 domain during infection, we investigated several bNAbs for their ability to neutralize the infectivity of NL-VA40774 (Fig. 6). NL-VA40774 was phenotyped in TZM-bl neutralization assays using a panel of bNAbs targeting various epitopes within Envelope. NL-VA40774 demonstrated sensitivity to multiple bNAbs targeting the CD4 binding site (CD4bs), V3-glycans, and membrane-proximal external region (MPER) of gp41 but was resistant to all V1/V2-glycan bNAbs. The following antibodies did not neutralize NL-VA40774 (i.e., had 50% inhibitory concentrations [IC_50_s] above 50 µg/ml): PG9, PG16, PGDM1400, CAP256-VRC26.25, PGT121, 8ANC195, 10E8, 17b, and 447-52D. A list of the antibodies tested and the epitopes that they recognize is provided in Materials and Methods.

**FIG 7:**
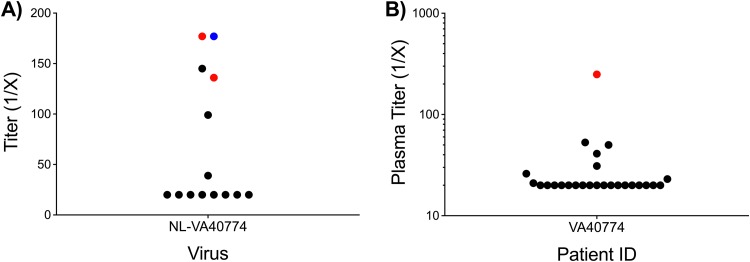
Neutralization characteristics of NL-VA40774 and VA40774 plasma. (A) Serum from 10 HIV-infected individuals and plasma from 4 HIV elite controllers, including VA40774, were tested for their ability to neutralize NL-VA40774. VA40774 (blue) and two chronically infected individuals (red) were able to significantly neutralize NL-VA40774, while the remaining 12 patients were not able to neutralize NL-VA40774 (black). Titers that were >3-fold above the murine leukemia virus (MuLV) control levels were considered significant. (B) A single plasma sample from VA40774 was screened for its ability to neutralize two panels of HIV-1 Env pseudoviruses in TZM-bl neutralization assays. VA40774’s plasma exhibited markedly low neutralizing activity. Of the 25 viruses tested, VA40774’s plasma was capable of neutralizing only the tier 1A strain SF162.LS (red). The following viruses were not neutralized by VA40774’s plasma (black): BaL.26, 6535.3, QH0692.42, SC422661.8, AC10.0.29, RHPA4259.7, REJO4541.67, WITO4160.33, TRO.11, THRO4156.18, CAAN5342.A2, PVO.4, TRJO4551.58, 25710-2.43, 398-F1_F6_20, CNE55, Ce1176_A3, X1632_S2_B10, Ce703010217_B6, 246-F3_C10_2, CH119.10, X2278_C2_B6, CNE8, and BJOX002000.03.2.

We also sought to determine whether plasma from 4 elite controllers or serum from 10 chronically infected HIV patients could neutralize NL-VA40774. Of the 4 plasma and 10 serum samples tested, only three were capable of neutralizing the infectivity of NL-VA40774 (Fig. 7A). One of the individuals who neutralized NL-VA40774 was VA40774, suggesting that this patient has a strain-specific response to his own primary isolate. The other two patients whose serum neutralized the infectivity of NL-VA40774 were chronically infected individuals. None of the elite controllers neutralized NL-VA40774 significantly above background except for VA40774. On the basis of its relative resistance to neutralization by both chronically infected individuals and several of the bNAbs tested in our panel, the characteristics of NL-VA40774 are most consistent with those of tier 2 viruses.

### Neutralization capacity of VA40774’s plasma.

Given that NL-VA40774 has an extremely long V1 domain and that it is completely resistant to neutralization by V1/V2-glycan-dependent antibodies, we sought to determine the extent to which plasma from VA40774 has neutralizing activity. We therefore tested the ability of VA40774’s plasma to neutralize the infectivity of 25 Env pseudoviruses in TZM-bl neutralization assays (Fig. 7B). The first panel included clade B viruses and sensitive tier 1 isolates in addition to the standard reference panel of clade B tier 2/3 viruses (27). The second panel consisted of the global reference panel of 12 isolates, which is predictive of breadth and potency against a more extended number of isolates (28). The plasma from VA40774 was capable of neutralizing only strain SF162.LS, a tier 1A clade B Envelope that is particularly sensitive to neutralizing antibodies. Thus, the neutralization capacity of VA40774’s plasma demonstrated the usual features of autologous neutralization and limited breadth (29).

### Escape from V1/V2-glycan-dependent antibodies is mediated by removal of the N160 glycan and not by V1 domain extension.

**FIG 8:**
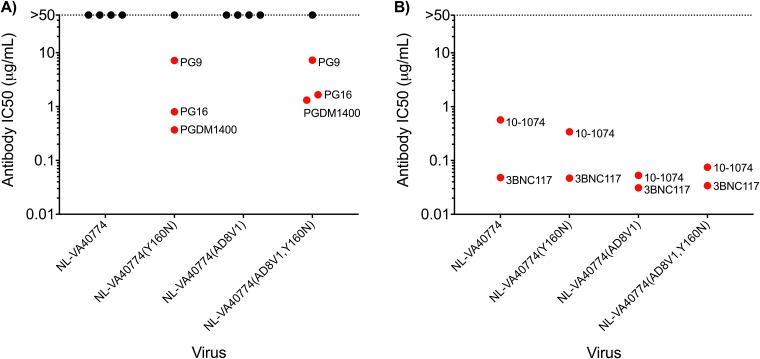
Role of the N160 glycan in determining the NL-VA40774 neutralization phenotype. NL-VA40774 and three NL-VA40774 recombinants were tested for their sensitivity to antibody neutralization. (A) Restoration of the N160 glycan rendered NL-VA40774 sensitive to neutralization by three of the four V1/V2-glycan-dependent bNAbs. In the presence of the N160 glycan, PG16 and PGDM1400 had slightly lower IC_50_s when the long V1 domain of NL-VA40774 was also present. Exchanging NL-VA40774’s long V1 domain with AD8V1 did not render the virus susceptible to neutralization by V1/V2-glycan bNAbs. Similarly, mutating the Y160 to N160 did not change the neutralization resistance of NL-VA40774 to CAP256-VRC26.25. (B) There was no significant difference in the IC_50_ of CD4bs bNAb 3BNC117 when NL-VA40774 was modified to contain AD8V1 or the N160 glycan or both. In contrast, the IC_50_ of V3-glycan bNAb 10-1074 decreased by 1 log when the short V1 domain of NL-AD8 was exchanged into NL-VA40774. The IC_50_ of 10-1074 was slightly lower in NL-VA40774(Y160N) than in NL-VA40774 but was slightly higher in NL-VA40774(AD8V1, Y160N) than in NL-VA40774(AD8V1).

Although NL-VA40774 was resistant to neutralization by all four tested V1/V2-glycan-dependent antibodies, this phenotype might be explained by the N160Y substitution in NL-VA40774 (30). We therefore sought to determine the extent to which neutralization by V1/V2-glycan-dependent antibodies could be restored by (i) reverting the N160 glycan, (ii) swapping the long V1 of NL-VA40774 for the short V1 of NL-AD8, or (iii) both (Fig. 8).

Restoration of the N160 glycan rendered NL-VA40774 sensitive to neutralization by three of the four V1/V2-glycan-dependent bNAbs, while exchange of the long V1 domain had no effect on neutralization by this class of antibodies (Fig. 8A). The only V1/V2-glycan bNAb that was unable to neutralize NL-VA40774(Y160N) was CAP256-VRC26.25, indicating that this antibody does not depend solely on the N160 glycan for neutralization.

We observed that the length of the V1 domain can modestly affect how the N160 glycan influences the broadly neutralizing antibody response. PG16 and PGDM1400 neutralized NL-VA40774(Y160N) slightly more potently than NL-VA40774(AD8V1, Y160N) (Fig. 8A). In addition, we tested the ability of a single CD4bs (3BNC117) and V3-glycan (10-1074) bNAb to neutralize these recombinant viruses (Fig. 8B). Reversion of Y160 to N160 rendered NL-VA40774 slightly more susceptible to neutralization by 10-1074 but not neutralization by 3BNC117. In the context of the short V1 domain recombinant NL-VA40774(AD8V1), however, restoration of the N160 glycan caused 10-1074 to neutralize virus slightly less potently. Taken together, these results indicate that the V1 domain length has a mild influence on the ability of the N160 glycan to modulate neutralization by certain classes of bNAbs.

### A long V1 domain mediates escape from some classes of broadly neutralizing antibodies.

Our results demonstrate that a long V1 domain does not serve as a mechanism of escape from V1/V2-glycan-dependent antibodies. We next investigated the extent to which the long V1 domain of VA40774 Env may mediate escape from members of several other classes of bNAbs, including CD4bs (3BNC117, VRC01, and N6), V3-glycan (10-1074, PGT121, and PGT128), gp120/41 (8ANC195), MPER (10E8, 4E10, 2F5), CD4i (17b), and V3 (447-52D) antibodies.

**FIG 9:**
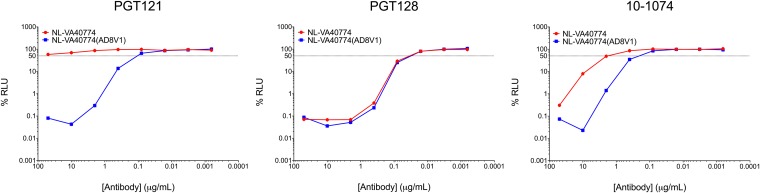
V1 domain length serves as a mechanism of escape from some V3-glycan broadly neutralizing antibodies. NL-VA40774 (red) and NL-VA40774(AD8V1) (blue) were tested for their capacity to be neutralized by V3-glycan broadly neutralizing antibodies (PGT121, PGT128, 10-1074). Exchanging the long V1 domain of NL-VA40774 with the short V1 domain of NL-AD8 rendered the virus at least 500-fold more susceptible to neutralization by PGT121 and 10-fold more susceptible to 10-1074. Exchange of the long V1 domain with a short V1 domain had no effect on neutralization by PGT128.

Swapping the long V1 domain of NL-VA40774 for the short V1 domain of NL-AD8 can substantially modify antibody IC_50_ in a class- and antibody-specific manner (Table 2). We observed the greatest fold change in IC_50_ for the V3-glycan-dependent class of bNAbs (Fig. 9). NL-VA40774(AD8V1) exhibited a 10-fold increase in sensitivity to neutralization by 10-1074 compared to NL-VA40774. Although an IC_50_ for PGT121 was above the limit of detection (>50 µg/ml) for NL-VA40774, the NL-VA40774(AD8V1) recombinant was potently neutralized by PGT121 with a concentration of approximately 0.1 µg/ml—a change of at least 500-fold. In contrast, there was no difference between the IC_50_s of PGT128 whether NL-VA40774 had a long or short V1 domain. There was little or no difference in the IC_50_s for the classes of bNAbs recognizing the CD4bs and MPER. The gp120/41 (8ANC195), CD4i (17b), and V3 (447-52) antibodies did not neutralize NL-VA40774 or NL-VA40774(AD8V1).

**TABLE 2:** Fold change in IC_50_ with a long versus short V1 domain

Monoclonal antibody	Epitope	IC_50_ (µg/ml)		Fold change^*a*^
		NL-VA40774	NL-VA40774(AD8V1)	
3BNC117	CD4bs	0.048	0.031	1.548
VRC01	CD4bs	0.470	0.410	1.146
N6	CD4bs	0.150	0.130	1.154
PG9	V1/V2-glycan	>50	>50	ND
PG16	V1/V2-glycan	>50	>50	ND
PGDM1400	V1/V2-glycan	>50	>50	ND
CAP256-VRC26.25	V1/V2-glycan	>50	>50	ND
10-1074	V3-glycan	0.567	0.053	10.698
PGT121	V3-glycan	>50	0.100	>500
PGT128	V3-glycan	0.030	0.030	1.000
8ANC195	gp120/41	>50	>50	ND
10E8	MPER	>50	>50	ND
4E10	MPER	29.820	33.040	0.903
2F5	MPER	12.620	14.680	0.860
17b	CD4i	>50	>50	ND
447-52D	V3	>50	>50	ND

a For several antibodies, the IC_50_ remained >50 µg/ml whether NL-VA40774 had a long or short V1 domain. In these cases, the fold change is listed as “ND” for “no difference.”

## DISCUSSION

In the majority of patients, untreated HIV-1 infection progresses to AIDS. Elite controllers are individuals who maintain normal blood cell counts and a low or undetectable viral load throughout decades of infection. Greater understanding of the mechanisms by which certain individuals control HIV-1 replication may provide insights into viral pathogenesis and inform the design of vaccines, therapeutics, and cure strategies. In this study, we set out to determine some of the virologic correlates of nonprogression by sequencing and functionally analyzing the HIV-1 primary isolate from an elite controller. Our analysis of HIV-1 isolated from VA40774 revealed a viral genome belonging to group M, clade B. This particular isolate had an unusual V1 domain in its Envelope at 49 amino acids in length, thus rendering it 25 amino acids longer than the clade B consensus strain. Moreover, the V1 domain in this isolate contained an additional two N-glycosylation sites and two cysteines relative to HXB2.

Each Envelope spike on the HIV-1 virion is a heterotrimeric complex consisting of three gp120 molecules noncovalently bound to three gp41 molecules (31). The mature spike, in its prefusion form, is tightly packed to limit the exposure of potentially antigenic epitopes to host neutralizing antibodies. HIV-1 infection results in the development of an extensive repertoire of antibodies against the viral surface Envelope glycoprotein gp120. Much of the antibody response in a given individual is mounted against the apex region (V1/V2 domains) of gp120, and such antibodies can result in strain-specific neutralization (32). These antibody responses, however, ultimately drive the evolution of the HIV-1 Envelope sequence in a manner that results in the loss of antibody potency as the autologous strain acquires resistance mutations. Broadly neutralizing antibodies, in contrast, target more highly conserved difficult-to-access regions of the Envelope trimer (33, 34).

While studies have shown that early sequence variation in V1 promotes escape from strain-specific antibody responses, it has only recently been reported that sequence variation in V1, particularly in the form of increases in V1 length, may counteract neutralization by bNAbs (32, 35). Three separate studies published between 2016 and 2017 reported a role for a long V1 domain in conferring escape from antibody neutralization (23, 36, 37). Those studies demonstrated that a long V1 region with extra cysteines and additional N-glycan sites can evolve within an infected individual to interfere with neutralization by bNAbs of the V3-glycan class. In our study, we observed that exchange of the long V1 domain with a short V1 domain is sufficient to confer neutralization susceptibility to PGT121 and cause a 10-fold decrease in the IC_50_ for 10-1074. Our results did not show an effect of V1 length on the ability of PGT128 to neutralize NL-VA40774, suggesting that a long V1 domain influences antibody neutralization in both class and antibody-specific manners.

Sequence adaptations in the HIV-1 genome that contribute to evasion from host defense mechanisms often come with a cost to viral fitness. We showed that the long V1 domain in NL-VA40774 contributes to virus evasion from the broadly neutralizing antibody response but that its extraordinary length attenuates infectivity. When exchanged into other virus strains, the unusually long V1 domain in NL-VA40774 dramatically decreased infectivity relative to the parental virus. van den Kerkhof et al. previously analyzed related Env sequences from a single patient that ranged in V1 length from 27 to 41 amino acids and found a statistically significant inverse correlation with virus replication over a 3-fold range (23). Although the V1 domain reported here caused markedly decreased infectivity of laboratory strains, we were surprised to find that NL-VA40774 was highly infectious and that reversion of the long V1 to a short V1 did not affect the infectivity of NL-VA40774. These results suggest that selective pressure for a long V1 domain drives sequence changes outside V1 to accommodate such an unusually long V1 domain. While it is clear that compensatory changes are required to facilitate the development of a long V1, it remains to be determined what these specific sequence alterations may be.

The long V1 region described here was almost certainly selected by the specificity of the antibody response. What we do not know is whether that antibody-driven selection occurred in the transmitting donor or in the VA40774 recipient. Unfortunately, sufficient samples were not available to determine whether the long V1 evolved in VA40774 or whether VA40774 may have been originally infected with an HIV-1 strain containing the unusually long V1 region. The usual mode of escape from the V3-carbohydrate class of potent broadly neutralizing antibodies is via mutation of the N332 glycan (38). However, patient VA40774 retains the N-glycan at position 332, which is preferred when antibodies of the V3-carbohydrate class are not present. We can speculate that antibodies of similar specificity but not dependent on N332 arose in the transmitting donor or in VA40774 to drive the unusual escape route of extreme elongation of V1. If the selection for a long V1 occurred in the transmitting donor, viral replication in VA40774 may not have been sufficient to allow reversion to a preferred V1 that was more normally sized. If the selection for the long V1 occurred in VA40774, the presence of memory B cells may prevent reversion to a V1 of more normal size but the level of these antibodies more than a decade later may be too low to allow detection because there are no longer Env sequences present to drive their expression to detectable levels.

In summary, we describe an elite controller whose HIV-1 strain has an extremely long V1 domain. Our results demonstrate that a long V1 domain promotes escape from some, but not all, of the V3-glycan bNAbs and that sequence accommodations within Envelope but outside V1 are necessary to prevent a reduction in viral infectivity. We hope that these findings contribute to the growing literature on escape from broadly neutralizing antibodies and provide insights into the potentially attenuating effects of such escape mutations.

## MATERIALS AND METHODS

### Patient inclusion.

The patient from this study was enrolled in an elite controller cohort at the University of Miami, Miami, FL. Scientific studies using specimens from these patients have been approved by the Institutional Review Board at the Bruce W. Carter Department of Veterans Affairs Medical Center at the University of Miami.

### Cell culture.

Patient and donor peripheral blood mononuclear cells (PBMCs) were cultured at a concentration of 1 × 10^6^ cells/ml in RPMI 1640 medium (RPMI) with 10% heat-inactivated fetal bovine serum (HI-FBS; Thermo Fisher Scientific) and 50 U/ml of interleukin-2 (IL-2) (R10-50U; R&D Systems). HEK293T and TZM-bl cells were cultured in Dulbecco’s modified Eagle’s medium (DMEM) with 10% HI-FBS (D10). The modified SupT1 cell lines were cultured in RPMI with 10% HI-FBS (R10). Each medium preparation was supplemented with 100 µg/ml of Primocin (InvivoGen).

### Viral outgrowth.

A single 30-ml blood draw was obtained from elite controller VA40774 and a healthy, HIV-negative donor. PBMCs were isolated by density gradient centrifugation with lymphocyte separation media (MP Biomedicals) in SepMate tubes (STEMCell Technologies). Cells were subsequently counted using a Beckman Coulter Z1 particle counter. Four million PBMCs from VA40774 were cultured overnight in R10-50U. Twenty-five million HIV-negative donor PBMCs were stimulated with phytohemagglutinin-M (PHA-M; Thermo Fisher Scientific) for 24 h to induce lymphoid cell activation and to promote a cellular environment permissive to HIV infection. After PHA-M incubation, both patient and donor PBMCs were centrifuged at 500 × g for 5 min, washed, and cocultured in R10-50U. The emergence of a primary HIV isolate was monitored using an antigen-capture kit to assess Gag-p24 levels in the supernatant (Advanced Bioscience Laboratories, Inc.).

### Viral RNA extraction and PCR amplification.

A single 1-ml aliquot of coculture supernatant containing 40 ng Gag-p24/ml was centrifuged at 16,000 × g for 2 h at 4°C to pellet virus. The supernatant was carefully removed, and the pellet was resuspended in phosphate-buffered saline (PBS) to reach a final volume of 140 µl. Viral RNA was extracted using a QIAamp viral RNA minikit (Qiagen) and immediately used to generate cDNA with the SuperScript IV first-strand synthesis system (Invitrogen) and UNINEF-7′ gene-specific primer (5′-GCACTCAAGGCAAGCTTTATTGAGGCTT-3′). Reverse transcription was carried out for 1.5 h at 50°C, and cDNA products were stored at –20°C.

The LANL tool “PrimerDesign-M” was used to design a primer set for amplification of near full-length ∼9-kb HIV-1 genomes (forward, 5′-GRGAACCCACTGCTTAAGCC-3′; reverse, 5′-GGTCTAACCAGAGAGACCCAGTACAG-3′). PCR was performed on 1 µl of cDNA using Phusion Hot-Start II High-Fidelity Master Mix (Thermo Fisher Scientific) in a single 50-µl reaction mixture. Cycling conditions for this PCR are 98°C for 3 min, followed by 30 cycles of 98°C for 10 s, 69°C for 15 s, and 72°C for 5 min, with a final extension at 72°C for 5 min.

### Near full-length HIV sequencing.

PCR products were analyzed on a 0.8% agarose gel at 120 V for 70 min. The ∼9-kb amplicon was subjected to gel extraction using a NucleoSpin Gel and PCR clean-up kit (Macherey-Nagel). Ten micrograms of gel-purified product was sent to DNA Link, Inc., for preparation of SMRTbell libraries according to the manufacturer’s instructions for 10-kb amplicons. After library preparation, amplicon quality was assessed on a Bioanalyzer and SMRT sequencing was performed on a PacBio RSII sequencer.

### Sequence analysis.

The raw data from the PacBio RSII were kindly analyzed at Eric Hunter’s laboratory using their published algorithm (39). The LANL “Gene Cutter” tool was used to parse the nucleotide sequences from the near full-length genome into the HIV-1 open reading frames. An amino acid alignment was generated for each of VA40774’s HIV-1 proteins against the HIV-1 clade B consensus, NL4-3, YU2, and ADA. Each protein sequence was carefully analyzed for rare polymorphisms, insertions, or deletions against these four reference strains and the entire 2016 LANL HIV-1 Sequence Compendium (40).

### Generation of recombinant virus.

A 3,246-bp stretch of DNA spanning VA40774’s HIV-1 *rev* and *env* was designed *in silico*, gene synthesized (GenScript), and cloned into NL4-3 using the EcoRI and XhoI restriction sites. The V1 domain of HIV-1 Envelope spans HXB2 amino acid positions 131 to 156. A set of oligonucleotides corresponding to HXB2-aligned positions 130 to 156 of NL4-3, NL-AD8, or NL-VA40774 was gene synthesized (GenScript). Each V1 domain was exchanged into the other two strains to generate all possible combinations of V1 recombinants. The integrity of the plasmids was confirmed by sequencing and restriction analysis.

HEK293T cells (American Type Culture Collection) were seeded in a T75 flask such that their confluence would reach ∼60% after 24 h. The cells were transfected with 5 μg of each virus-encoding plasmid using jetPRIME (Polyplus-transfection SA), and complete medium changes were performed at 24 and 48 h posttransfection. At 72 h, virus-containing supernatant was harvested and clarified by two rounds of centrifugation (500 × g for 5 min and 2,000 × g for 10 min). The concentration of each virus stock was measured using a Gag-p24 antigen-capture kit (Advanced Bioscience Laboratories, Inc.). All viruses were stored at –80°C prior to experimentation.

### TZM-bl infectivity and neutralization assays.

The infectivity and neutralization assays were performed as previously described (41). Briefly, to measure infectivity, each virus stock was diluted to a concentration of 80 ng Gag-p24/ml and 2-fold serial dilutions were performed eight times, in triplicate, in a 96-well plate. One hundred microliters of each diluted virus stock was transferred to a 96-well plate containing 10,000 TZM-bl cells per well and stored in a 37°C incubator with 5% CO_2_. After 48 h, viral infectivity per nanogram of Gag-p24 was determined based on luciferase activity using a Britelite plus kit (PerkinElmer). The neutralization assay was performed similarly, except that a single concentration of virus was used that gave results corresponding to approximately 150,000 relative light units (RLU) after 48 h. Each monoclonal antibody was tested at a primary concentration of 50 µg/ml and subjected to 5-fold titrations seven times. The antibody and virus were incubated together for 1 h prior to the addition of TZM-bl cells. The panel of antibodies tested in the neutralization assays recognized the CD4 binding site (3BNC117, VRC01, VRC07-523, N6), V1/V2-glycans (PG9, PG16, PGDM1400, CAP256-VRC26.25), V3-glycans (10-1074, PGT121, PGT128), gp120/41 (8ANC195), the membrane-proximal external region of gp41 (10E8, 4E10, 2F5), the CD4-induced (CD4i) binding site in gp120 (17b), and V3 (447-52D).

### Coreceptor usage and replicative capacity.

Two modified SupT1 cell lines expressing either CXCR4 or CCR5 were kindly provided by James Hoxie (42). Three million cells (SupT1-CCR5 or SupT1-CXCR4) were infected with 10 ng Gag-p24 of each HIV-1 stock at a density of 5 × 10^5^ cells/ml. The cells were washed with PBS the following day, and a complete medium change was performed to remove excess virus from each infection condition. The cell cultures were split 1:3 every other day, and the supernatants were clarified of cellular debris by centrifugation at 500 × g for 5 min. Samples were stored at –20°C prior to analysis of supernatant concentrations of Gag-p24.

### HLA, KIR, and CCR5 genotyping.

DNA was extracted from VA40774’s PBMCs using a DNeasy blood and tissue kit (Qiagen). A qualitative PCR was performed to determine whether *ccr5* in VA40774 had the 32-bp deletion associated with elite control (43). Four micrograms of DNA was sent to Histogenetics LLC for SMRT sequencing of the HLA and KIR alleles.

### Data availability.

Sequences for the near full-length VA40774 HIV-1 isolate and each of the nine ORFs are available at GenBank under accession number MK499378.
